# DNA Damage, DNA Repair, and Cancer: Second Edition

**DOI:** 10.3390/ijms242316835

**Published:** 2023-11-28

**Authors:** Kazuhiko Kuwahara

**Affiliations:** Department of Diagnostic Pathology, Kindai University Hospital, Osaka 589-8511, Japan; kazukuwa@med.kindai.ac.jp

Following our first Special Issue, we are pleased to present this Special Issue in the *International Journal of Molecular Sciences*, titled ‘DNA Damage, DNA Repair, and Cancer: Second Edition’. During mitosis, one cell divides into two cells with the same genetic information. DNA, the basic unit of inheritance, should be faithfully transmitted into two daughter cells; however, DNA is known to be damaged at 10,000 to 1,000,000 locations per cell every day. Not only external stimulus such as radiation but also endogenous stresses including oxidation are associated with the increase in the risk of cancer due to DNA damage. There are various DNA repair mechanisms that protect the DNA in living cells by removing damaged lesions. If cells respond to DNA damage, DNA repair mechanisms are promptly activated throughout the different cell cycle stages. Understanding this molecular mechanism teaches human beings the right ways to prevent cancer development.

Additionally, recent research progress has facilitated cancer treatment using dysfunctional DNA repair. Most importantly, PARP inhibitors have been evaluated and developed in BRCA-related tumors such as ovarian, breast, prostate and pancreatic carcinomas. These drugs are useful for cancer treatment via the use of the so-called synthetic lethality. Several clinical trials have demonstrated that they could improve patients’ survival in homologous recombination deficiency with or without *BRCA* mutations compared to non-homologous recombination deficiency patients.

This Special Issue is a compilation of seven research manuscripts and reviews covering all aspects of DNA repair mechanisms and its related disorders. The first five original articles investigate the following: the effect of a DNA-PK inhibitor in chronic and acute myeloid leukemia cells [1]; marked effects of RPA inhibition on the cell cycle and the DNA damage response [2]; gene expression profiles after the treatment of TOP1 or TDP1 inhibitors [3]; a novel mechanism of VRK1 kinase activity in histone modification [4]; and the biological impact of the combined treatment of panobinostat and melphalan in multiple myeloma [5]. Moreover, the two reviews in this Special Issue address the initial integrations of Hepatitis B virus to hepatocyte to explain HBV-associated oncogenesis [6] and the therapeutic strategy of PARP-1 inhibition for ETS-expressing tumors [7].

Overall, this Special Issue focuses on basic research of DNA repair utilizing well-established techniques to obtain novel information. In addition, some papers discuss potential candidates for the medical treatment of malignant tumors. However, despite promising clinical results, drug resistance remains a challenge. To overcome adverse outcomes and long-term risks of complications further comprehensive studies on basic DNA repair mechanisms are needed.
